# Exposure to Magnetic Fields Changes the Behavioral Pattern in Honeybees (*Apis mellifera* L.) under Laboratory Conditions

**DOI:** 10.3390/ani12070855

**Published:** 2022-03-29

**Authors:** Paweł Migdał, Ewelina Berbeć, Paweł Bieńkowski, Mateusz Plotnik, Agnieszka Murawska, Krzysztof Latarowski

**Affiliations:** 1 Bee Division, Department of Environment, Hygiene and Animal Welfare, Wroclaw University of Environmental and Life Sciences, 25 C.K. Norwida St., 51-630 Wroclaw, Poland; ewelina.berbec@upwr.edu.pl (E.B.); mateuszplotnik98@gmail.com (M.P.); agnieszka.murawska@upwr.edu.pl (A.M.); 2 Telecommunications and Teleinformatics Department, Wroclaw University of Science and Technology, 27 Wybrzeze Wyspianskiego St., 50-370 Wroclaw, Poland; pawel.bienkowski@pwr.edu.pl; 3 Department of Human Nutrition, Wroclaw University of Environmental and Life Science, 25 C.K. Norwida St., 51-630 Wroclaw, Poland; krzysztof.latarowski@upwr.edu.pl

**Keywords:** honeybee, magnetic field, electromagnetic field, behavior, insect, social insects, invertebrates

## Abstract

**Simple Summary:**

Electrically powered devices and power lines generate electromagnetic fields. Technological development has resulted in environmental pollution with anthropogenic electromagnetic fields. One of its components is the magnetic field. Its impact on living organisms is still under investigation, but there are reports suggesting that the direction of change is negative. Pollinators are very important for the environment and are also exposed to this factor. In this study, we wanted to investigate the impact of magnetic field exposure on the behavior of one of the key pollinators: the honeybee. The frequency of the tested field corresponded to those present under high voltage lines, as honeybees often forage in these areas. The results showed that the magnetic field caused a distribution in behavioral patterns, which may have a direct impact on foraging efficiency and pollination success.

**Abstract:**

Earth’s magnetic field (MF) plays an important role for many species, including the honeybee, in navigation. Nowadays, much larger alternating fields are emitted by miscellaneous electric infrastructure components, such as transformers and power lines, and the environment is therefore polluted by an anthropogenic electromagnetic field, though little is known regarding its impact on living organisms. The behavior of animals is the first and easiest way to establish the impact of stress. It shows if the animal can detect the exposure and react to it. To investigate this, one-day-old bees were exposed to a 50 Hz magnetic field of induction at 1 mT and 1.7 mT for 10 min, 1 h, and 3 h under laboratory conditions. All groups exposed to the magnetic field showed differences in behavioral patterns. What is more, they presented a behavior absent in the control: loss of balance. There were differences, both in the ratio of behaviors and in the number of bouts—exposed bees more often changed behavior. Occurrence of differences is an indication of the reaction of the honeybee organism to the magnetic field. Loss of balance is a disturbing symptom, and behavior changes indicate a disturbance of the honeybee by the electromagnetic field.

## 1. Introduction

Pollination forms the basis of complex ecological systems and is essential for agricultural production. It is estimated that 75% of main crops need animal pollinators [1]. The financial benefits of pollinators are estimated at USD 153 billion, or 9.5% of the total value of the world food market [2]. Bees are the main pollinators in terrestrial ecosystems, and it is through them that both biological and genetic diversity is maintained [3,4]. An invaluable role in this process can be attributed to the honeybee (*Apis mellifera* L.). Nowadays, the honeybee population faces many harmful factors and stressors: parasites and pests, such as *Nosema ceranae* and *Varroa destructor* mite [5,6,7]; monocultures [8], plant protection products [9]; environmental pollution [10]; climate changes [11]; and artificial electromagnetic fields [12,13].

An electromagnetic field (EMF) consists of two components: an electric field (EF), with intensity expressed in V/m, and a magnetic field (MF), with magnetic induction expressed in tesla (T).

Natural and anthropogenic sources of EMF in the environment can be distinguished. Natural EF can be visible as storms and lighting prompted by the interaction of electrically charged air masses [14]. Natural MF is the cause of magnetosphere occurrence. The magnetosphere is the space above the ionosphere, around the Earth. It extends tens of thousands of kilometers into space and forms a shield that deflects streams of high-energy solar particles (solar wind). It protects the ozone layer and thus the planet’s surface from overexposure to ultraviolet radiation. The absence of this buffer zone would be disastrous for life on the planet’s surface. The source of Earth’s magnetic field is due to the structure of the planet’s interior. It is assumed that, due to the friction between the inner nucleus and the outer liquid nucleus, an electrostatic charge is created. The value of the magnetic field induction depends on the latitude and is, for example, 65 μT at the magnetic poles and 27 μT at the magnetic equator [15]. As electric charge movement induces electromagnetic radiation, every electrical device generates EMF. Radio-frequency radiation is intentionally generated and targeted from space satellites and ground localized transmitters for communication. The frequency of the generated EMF depends mainly on device type, but intensity and magnetic induction mostly depends on the distance from the device [16,17].

Migratory animals are most affected by magnetic fields. As well as birds, there are many migratory animals, including dolphins [18], sea turtles [19,20], salmon [20], or insects such as the monarch butterfly (*Danaus plexippus* L.) [21]. Over the years, these animals and many others have developed their own navigation systems that allow them to reach their destinations, often thousands of kilometers away. A magnetic sense has also been discovered in invertebrates: insects, including bees and wasps [15], and mollusks [22,23]. Honeybees (*Apis mellifera* L.) have the ability to remember the position of a food source in relation to magnetic field lines. This information is communicated to other members of the hive through waggle dances [15,24]. It was suggested that artificial MF greater than 500 µT can disturb honeybee magnetic navigation [17,25].

EMF is a very common factor in the environment, but its influence on living organisms is still poorly understood. It was observed that EMF affects biological systems [26]. Electric power transmission lines generate EMF with a frequency of 50 Hz or 60 Hz, depending on local regulations. Therefore, EMF of this frequency is very common in terrestrial environments, including areas where bees are foraging and hives are situated. As early as 1981, Greenberg et al. [12] observed significant disturbances in bee colonies under EMF exposure. Hives were long-term exposed to a 7 kV/m 59 µA and 85 µA field (corresponding to a field under high-voltage power lines). This led to hive entrances aberrant propolisation, queen loss, and a decrease of capped brood (while the number of eggs and larvae were normal). Additionally, winter survival was decreased in EMF-exposed colonies. These findings led to a recommendations in the USA to not keep bee colonies under power lines [17].

As honeybees, unlike most other farm animals, live in an uncontrolled environment, both the life of the individual honeybee and that of the whole colony is dependent largely on themselves: on foraging success, parasite control, raising offspring, seasonal changes in colony structure, and answers to other environmental challenges. Therefore, the ability to proceed with all complex and connected activities is necessary to keep the colony alive. What is more, all of this translates into pollination success, making it crucial for the existence of the environment as we know it. The results of behavioral studies are well visible and have direct impacts, both on individual life or death and on whole colony integrity, even when mechanisms of behavior determination have not yet been fully investigated. This is why behavioral studies on honeybee are so important. Behavioral changes are investigated as a response to stressors, such as parasites and diseases [27] or pesticides [28].

The aim of this study was to investigate behavioral changes after exposure to 50 Hz MF of 1 and 1.7 mT magnetic induction and various exposure time variants in honeybee workers.

## 2. Materials and Methods

### 2.1. Research Material

In the experiment, one-day-old worker honeybees were used. For research, colonies of *Apis mellifera carnica* were chosen as a brood source. Frames with a brood capped at 20 days of age were taken from the hive to an incubator with a temperature of 34 °C and humidity of 70–80%. Newly emerged worker bees were gently carried by hand to wooden cages; 100 bees to one cage. Each group consisted of 10 cages. Cages with available bee sugar syrup 1:1 (*w*:*v*) were maintained in an incubator for 24 h. Worker bees were fed ad libitum.

### 2.2. Exposure to Magnetic Field (MF)

A uniform alternating 50 Hz magnetic field was generated in a solenoid with a diameter of 350 mm and a length of 350 mm. The solenoid was supplied by a stabilized and controlled mains-powered sinusoidal current source. The distribution of magnetic induction in the testing area was measured and controlled using an ESM-100 S/N 972153 m calibrated by an accredited calibration laboratory, AP-078 (calibration certificate LWiMP/W/85/21). The measurements were carried out by the accredited testing laboratory, LWiMP AB-361. Induction non-uniformity in the whole measuring area did not exceed 5%. Due to the fact that the inner and outer surfaces of the coil winding were electrostatically shielded, the 50 Hz electric field inside the coil did not exceed 100 V/m.

Cages with dimensions of 200 mm × 150 mm × 70 mm were taken to the magnetic field (MF) emitter, and then emission began. There was only one cage in the emitter during exposure. There were three different times of exposure and two magnetic field intensities (6 experimental groups in total and 1 control group).

Times of exposure:

- 10 min—time responding to a short flight, such as to collect water or for defecation
- 1 h—the mean time of a forage flight
- 3 h—long forage time, necessary to bring a heavy load over a long distance

Magnetic field intensities:

- 1 mT
- 1.7 mT

### 2.3. Behavioral Analysis

Immediately after exposure, six randomly chosen bees from each cage were taken to a glass container with a height of 15 cm and a diameter of 20 cm. Bees were filmed for 300 ± 1 s using a SONY HDR-CX240E camera (Sony Mobile Comunications, Lund, Sweden). During the whole experiment, 420 worker bees were used. The videos were later analyzed offline. Seven types of behavior were distinguished:

- Walking—walking on the base surface or walls of the container
- Flight—short episodes of lifting up by wing movement
- Body cleaning—cleaning own body by legs
- Contact between individuals—any kind of near contact between bees, including touching antennas, trophallaxis
- Wings movement—the rapid movement of wings, used for ventilation, does not cause lifting up
- Stillness—staying motionlessly
- Loss of balance—bees from the walls of container fall and land on the bottom of the container upside down.

All analyzed types of behavior were mutually exclusive. A 300 s sample from each video was analyzed three times, one per bee. For analysis, we chose the mean total time per bee (how much time bees from one group spent on the behavior) and the number of individual behavior occurrences (how many times during the observation individuals from the group displayed the behavior). Each behavior was immediately marked from its occurrence to its end. The end of one behavior was the start time of another behavior. The recording of the bees came immediately after the end of exposure to the magnetic field.

### 2.4. Statistical Analysis

Data were analyzed in RStudio (R Core Team), using packages “dplyr”, “tidyr”, “agricolae”, and “ggplot2” for visualizations. A Shapiro–Wilk test was used to verify the normality of data distribution, a Kruskal–Wallis test with Holm correction was used for multiple comparisons, and α = 0.05 was used to check the significance of differences between groups.

## 3. Results

**Figure 1:**
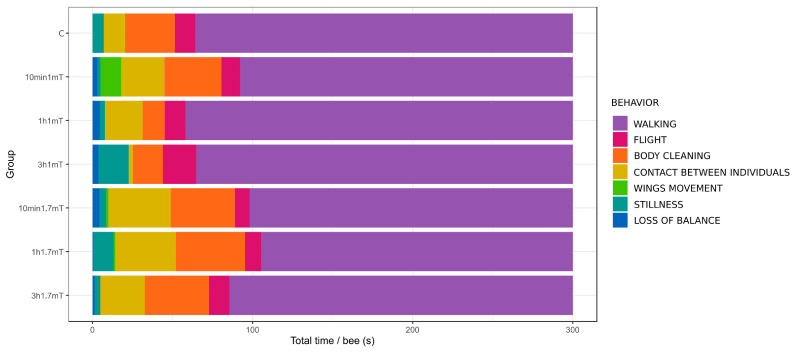
Mean time spent on behavior per bee in 5 min trial. The name of the group is a combination of time of exposure and intensity of the magnetic field, for example, 10 min 1 mT—bees were exposed for 10 min at 1 mT magnetic field intensity.

**Figure 2:**
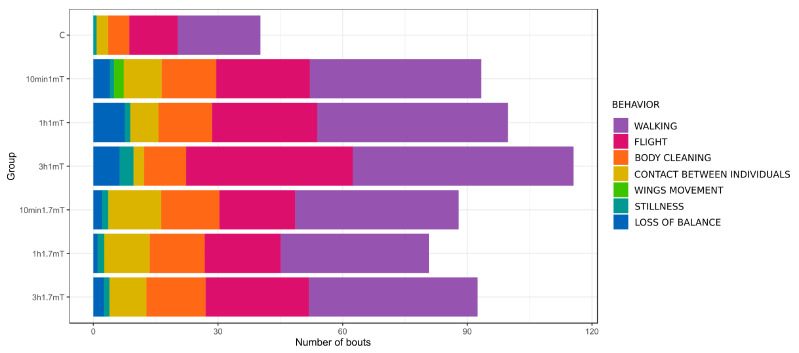
Mean number of bouts per bee. All exposed groups present visible differences from the control, with a much higher total number of bouts. Details under Figure 1.

**Figure 3:**
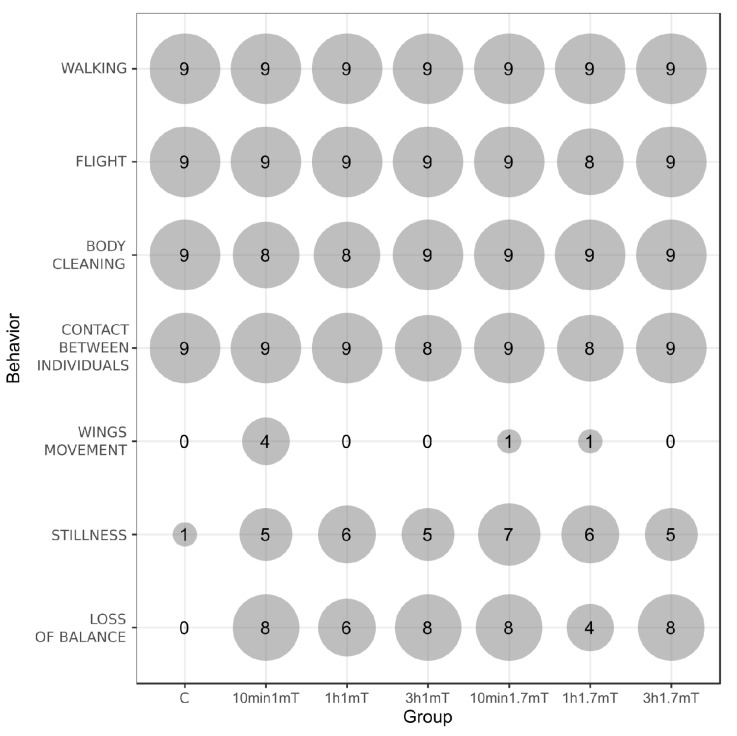
Number of bees per group that presented particular behaviors (areas of circles correspond to values). Total number of observed individuals were 9 in each group. Details under Figure 1.

Detailed data of total time spent on behaviors per bee and the mean number of behavior occurrences are presented in Table 1 and Table 2. Schemes of behavior patterns are visualized in Figure 1 and Figure 2. Figure 3 shows the number of bees that presented particular behavior.

Four out of the seven distinguished behaviors were presented by almost all observed individuals in all groups: walking, flight, body cleaning, and contact between individuals (Figure 3). Walking was the main behavior within all groups, as this presented the highest values, both in total duration time and number of occurrences. All exposed groups, except 1 h 1.7 mT, had significantly more occurrences of walking compared to the control (Table 2), but total duration time presented no differences (Table 1).

In the case of total time spent on flight, body cleaning, and contact between individuals, all of the EMF-exposed groups presented significant differences compared to the control, but there were some differences between exposed groups: group 3 h 1 mT had a lower value of time spent on contact between individuals compared to all other treatment groups, and less time spent on flight, but only compared to the 10 min 1.7 mT group (Table 1). Average times spent on body cleaning differed slightly between groups, but varied between individuals, so the differences were not statistically significant.

If we consider the number of behavior occurrences in the case of flight, body cleaning, and contact between individuals, the differences are more visible. Group 3 h 1 mT presented significantly more occurrences of flight compared to the control. More occurrences of body cleaning than the control were seen in all groups exposed to the 1.7 mT field, as in the case of contact between individuals, but this time the 10 min 1 mT group also presented significantly higher values.

All of the behaviors present in the control were also present in all groups exposed to MF, but there were behaviors present in the treatment groups that were absent or very poorly inherent in the control (Figure 3). When all individuals in the group did not present behavior, the value was marked as “NO” (not observed) and excluded from statistical analysis as non-numerical data (Table 1 and Table 2). Wing movement was generally the rarest behavior and was absent or very rare in most of the groups, including the control. Only in the 10 min 1 mT group was the situation different—wing movement was only slightly often. Stillness was presented in the control by only 1 bee, while in each treatment group this behavior was quite often and presented by more than half of the observed individuals, but in total time spent on this behavior and the number of bouts, significant differences did not appear. The most time on this behavior was seen in groups 3 h 1 mT and 1 h 1.7 mT. Loss of balance was a behavior totally absent in the control, but present and quite common in all exposed groups.

Group 10 min 1 mT, on average, spent a significant amount of time on wing movement, but as the behavior was poorly presented in other groups and was not very often seen in this group, it can be considered as marginal behavior. A significant amount of time was spent on stillness in groups 3 h 1 mT and 1 h 1.7 mT, group 3 h 1 mT spent very little time on contact between individuals, and group 1 h 1 mT spent only a little time on body cleaning. Groups exposed to 1 mT MF presented more appearances of loss of balance behavior than groups exposed to 1.7 mT MF. All of these differences were well visible on plots and were distinctive for the treatment groups, but not significant statistically.

All groups exposed to MF presented significant differences in behavior compared to the control. In particular, the number of bouts mostly diverged and a much higher number occurred after exposure in all groups, considering both single behaviors and total number of bouts. Another well-visible effect of exposure is the appearance of loss of balance—a behavior absent in the control group.

## 4. Discussion

In this study, the effects of EMF on honeybee behavior were evaluated. Through observation of bee behavior after MF exposure, the behavioral patterns of young workers exposed to 1 mT and 1.7 mT MF for 10 min, 1 h, and 3 h were assessed. Exposure to MF proved to significantly affect the behavioral patterns. It is difficult to determine the direction of changes with increasing time or intensity of exposure, while each group presented individual behavioral patterns. In many cases, however, all exposed groups had similarities significantly distinguishing them from the control.

Well-visible differences occurred in the number of behavior occurrences: exposed groups presented a much higher number of bouts compared to the control, while the ratio of behavior duration did not differ so significantly. This means that bees changed behavior significantly more often, and the frequency of changes was higher. Considering the number of bouts, the impact of an electric field (EF) might be distinguished as oppositely directed: given exposures to a 5 kV/m, 11.5 kV/m, 23 kV/m, and 34.5 kV/m field by 1 h, 3 h, 6 h, and 12 h, it could be seen that there was a slight general tendency for a decrease in the number of behavior occurrences and an increase of mean duration time [29,30].

An alternated mobility associated with MF influence has been demonstrated for invertebrates. Shepherd et al. [16], by tethered flight experiments on honeybees, showed that during exposure to 0.1 mT, 1.0 mT, and 7.0 mT MF, wingbeat frequency was increased, with a greater effect at higher exposure levels. The influence of MF on wingbeat frequency has also been demonstrated in locust [31]. A static 50 mT MF modulated the motor behavior of *Tenebrio molitor* and *T. obscurus*, but interestingly, even in such closely related species, the effects were remoted [32]. In our studies, the mobility-associated behaviors were walking and flight. Neither MF-exposure variant showed alterations in the ratio of total time spent on these behaviors. The differences were, however, noticeable in the number of bouts, where exposure prompted an increase in the number of occurrences. Therefore, a given behavior lasted much shorter but occurred more often.

Clearly differentiating from the control is the appearance of loss of balance behavior after MF exposure. In each group exposed to MF, this behavior was presented by more than half of the observed individuals, except in the 1 h 1.7 mT group, where, in 4 out of 9 individuals, this behavior occurred, which is still almost half. In general, this behavior both occurred more often and took more time in the behaviors ratio in bees exposed to 1 mT compared to 1.7 mT, while the time of exposure did not have such a visible impact. However, these differences are not statistically significant as wide variations between individuals occurred. Similar behavior, described as problems with movement coordination, trembling, tumbling, and lying upside down, has been observed as a result of poisoning after oral administration of neonicotinoids pesticides [28,33,34]. Williamson et al. [28] also observed this behavior in the control, but it intensified after pesticide exposure. In research on the impact of 50 Hz EMF on honeybee behavior, but with natural magnetic components and generated increased electric components, such behavior was not noted, either in the control or in the EF-exposed groups [29,30].

In our study, only unrestrained behavior was observed in non-demanding and simple environments. This proves that, even if we do not enforce specific reactions on honeybees, the impact of MF on behavior is noticeable. Other studies have also focused on the analysis of behavior, such as reactions to stimuli and success in task achievement, under MF exposure. It was demonstrated that MF of magnitudes 100 µT and 1000 µT reduce honeybee olfactory learning. Bees were exposed to MF for 1 min, and then immediately proboscis extension response (PER) was examined; this was repeated five times. Bees exposed to 100 µT and 1000 µT MF had a significantly lower level of response compared to the control. Bees exposed to 20 µT MF were not so disrupted. This led to the conclusion that MF can disturb foraging efficiency, which was also evaluated in field experiments. A zone of 100 µT MF exposure was situated between the hive entrance and the feeder in a restricted area. During 15 min of experiment duration, fewer bees flew out from the hive; a decrease in the number of bees returning to the hive was also observed, compared to the non-exposed control, but generally bees that successfully reached the feeder returned to the hive. Therefore, a decrease in forage efficiency as a result of MF influence was displayed [17]. Long-term exposure to MF (17 h) was also demonstrated to have an impact on aversive learning and aggression levels. In the sting expression response (SER) experiment, exposure to 100 µT and 1000 µT MF reduced aversive learning. The aggression level was investigated as a reaction to bees from foreign colonies; bees after 100 µT MF exposure presented much higher aggression scores [35].

Honeybees in the environment can be exposed to different stressors at any one time. The results of their coexistence can be difficult to predict. As described above, magnetic field exposure causes changes in honeybee wingbeat frequency and reduced olfactory learning. Interestingly, it has been demonstrated that simultaneous exposure to low-dose neonicotinoids and MF can attenuate this effect [36]. Investigating the impact of EMF in combination with other potentially harmful factors is an interesting issue for further studies.

## 5. Conclusions

Exposure to a 1 mT and 1.7 mT magnetic field significantly affected the behavioral pattern of young honeybee workers. It is difficult to denote the direction of the changes, but it was clearly shown that both time of exposure and the magnetic induction of the field makes a difference. The number of behavior bouts increased after all the tested exposures.

## Figures and Tables

**Table 1:** Mean total time per bee (s) ±SD, different letters within same behavior—significant differences (Kruskal–Wallis test, *p* < 0.05), NO—not observed. NO values per group were not included in statistical analysis in any way.

Name of Behavior	Groups and Time of Exposure							*p* (chisq)
	Control	1 mT			1.7 mT			
		10 min	1 h	3 h	10 min	1 h	3 h	
Walking	235.7 ± 30.7	207.7 ± 39.6	241.8 ± 24.9	235.1 ± 33.0	201.7 ± 42.6	194.7 ± 60.9	214.5 ± 42.0	0.094
Flight	12.9 ± 9.0 ^ab^	11.9 ± 12.3 ^ab^	13.2 ± 6.0 ^ab^	21.1 ± 8.1 ^a^	9.4 ± 9.1 ^b^	10.1 ± 9.8 ^ab^	12.8 ± 7.6 ^ab^	0.035
Body cleaning	31.1 ± 37.2	35.5 ± 29.0	13.7 ± 12.1	18.7 ± 29.0	40.1 ± 32.5	43.2 ± 64.9	40.1 ± 49.4	0.239
Contact between individuals	13.2 ± 13.4 ^ab^	27.0 ± 22.0 ^b^	23.5 ± 19.5 ^b^	2.6 ± 2.6 ^a^	38.8 ± 28.5 ^b^	37.8 ± 33.3 ^b^	27.8 ± 20.4 ^b^	0.0019
Wings movement	NO	13.0 ± 37.3	NO	NO	1.4 ± 4.2	1.0 ± 3.0	NO	0.558
Stillness	7.1 ± 21.4	1.9 ± 2.8	3.17 ± 5.4	18.9 ± 29.8	4.2 ± 6.0	12.6 ± 18.3	3.4 ± 4.2	0.348
Loss of balance	NO	3.1 ± 3.6	4.7 ± 11.7	3.7 ± 4.2	4.4 ± 10.9	0.6 ± 0.8	1.4 ± 1.0	0.289

chisq—the chi-squared distribution.

**Table 2:** Mean number of bouts ± SD, different letters within same behavior—significant differences (Kruskal–Wallis test, *p* < 0.05), NO—not observed. NO values per group were not included in statistical analysis in any way.

Behavior	Groups and Time of Exposure							*p* (chisq)
	Control	1 mT			1.7 mT			
		10 min	1 h	3 h	10 min	1 h	3 h	
Walking	19.9 ± 6.5 ^a^	41.2 ± 17.0 ^b^	45.9 ± 9.3 ^b^	53.1 ± 12.4 ^b^	39.3 ± 13.5 ^b^	35.7 ± 15.2 ^ab^	40.6 ± 12.7 ^b^	0.00033
Flight	11.7 ± 7.4 ^a^	22.6 ± 22.3 ^ab^	25.3 ± 11.7 ^ab^	40.1 ± 14.9 ^b^	18.2 ± 17.0 ^a^	18.3 ± 16.3 ^a^	24.9 ± 14.6 ^ab^	0.0053
Body cleaning	5.1 ± 3.6 ^a^	13.1 ± 9.8 ^ab^	12.9 ± 7.0 ^ab^	10.1 ± 6.5 ^ab^	14.0 ± 5.8 ^b^	13.2 ± 3.5 ^b^	14.2 ± 5.8 ^b^	0.021
Contact between individuals	2.8 ± 1.7 ^a^	9.1 ± 6.7 ^b^	6.8 ± 3.5 ^ab^	2.6 ± 2.0 ^a^	12.7 ± 5.9 ^b^	10.9 ± 5.6 ^b^	8.9 ± 4.2 ^b^	7.8 × 10^−5^
Wings movement	NO	2.3 ± 4.3	NO	NO	0.1 ± 0.3	0.1 ± 0.3	NO	0.56
Stillness	0.8 ± 2.3	1.0 ± 1.3	1.3 ± 1.7	3.3 ± 5.2	1.4 ± 1.2	1.6 ± 1.5	1.3 ± 1.7	0.49
Loss of balance	NO	4.0 ± 5.4	7.6 ± 20.1	6.3 ± 6.6	2.1 ± 3.7	1.0 ± 1.7	2.6 ± 2.0	0.054
TOTAL	40.2 ± 12.4 ^a^	93.3 ± 36.7 ^b^	99.8 ± 19.3 ^b^	116.0 ± 27.0 ^b^	87.9 ± 25.8 ^b^	80.8 ± 31.8 ^ab^	92.4 ± 26.5 ^b^	0.00021

chisq—the chi-squared distribution.

## Data Availability

The datasets generated and/or analyzed during the current study are available from the corresponding author on reasonable request.
